# Association between Measures of Women’s Empowerment and Use of Modern Contraceptives: An Analysis of Nigeria’s Demographic and Health Surveys

**DOI:** 10.3389/fpubh.2016.00293

**Published:** 2017-01-09

**Authors:** Ibitola O. Asaolu, Chioma T. Okafor, Jennifer C. Ehiri, Heather M. Dreifuss, John E. Ehiri

**Affiliations:** ^1^Department of Health Promotion Sciences, Mel and Enid Zuckerman College of Public Health, University of Arizona, Tucson, AZ, USA; ^2^Synergy America, Incorporation, Duluth, GA, USA; ^3^Department of Physiology, College of Medicine, University of Arizona, Tucson, AZ, USA

**Keywords:** women’s empowerment, gender equality, Nigeria, contraceptives, family planning, sub-Saharan Africa, population and development

## Abstract

**Background:**

Women’s empowerment is hypothesized as a predictor of reproductive health outcomes. It is believed that empowered girls and women are more likely to delay marriage, plan their pregnancies, receive prenatal care, and have their childbirth attended by a skilled health provider. The objective of this study was to assess the association between women’s empowerment and use of modern contraception among a representative sample of Nigerian women.

**Methods:**

This study used the 2003, 2008, and 2013 Nigeria Demographic and Health Survey data. The analytic sample was restricted to 35,633 women who expressed no desire to have children within 2 years following each survey, were undecided about timing for children, and who reported no desire for more children. Measures of women’s empowerment included their ability to partake in decisions pertaining to their healthcare, large household purchases, and visit to their family or relatives. Multivariable regression models adjusting for respondent’s age at first birth, religion, education, wealth status, number of children, and geopolitical region were used to measure the association between empowerment and use of modern contraceptives.

**Results:**

The proportion of women who participated in decisions to visit their relatives increased from 42.5% in 2003 to 50.6% in 2013. The prevalence of women involved in decision-making related to large household purchases increased from 24.3% in 2003 to 41.1% in 2013, while the proportion of those who partook in decision related to their health care increased from 28.4% in 2003 to 41.9% in 2013. Use of modern contraception was positively associated with women’s participation in decisions related to large household purchases [2008: adjusted OR (aOR) = 1.15; 95% CI = 1.01–1.31] and (2013; aOR = 1.60; 1.40–1.83), health care [2008: (aOR = 1.20; 1.04–1.39) and (2013; aOR = 1.39; 1.22–1.59)], and visiting family or relatives [2013; aOR = 1.58; 1.36–1.83]. The prevalence of modern contraceptive use among women with need for contraception increased marginally from 11.1% in 2003 to 12.8% in 2013.

**Conclusion:**

Although there were marked improvements in all measures of women’s empowerment between 2003 and 2013 in Nigeria, the use of modern contraceptives increased only marginally during this period. Beyond women’s participation in household decision-making, further research is needed to elucidate how measures of women’s empowerment interact with cultural values and health system factors to influence women’s uptake of contraceptives.

## Introduction

Safe motherhood has long been a major focus of many philanthropic, national, and international agencies (1). Despite numerous interventions aimed at addressing maternal and child health, many low- and middle-income countries continue to report high rates of mortality among women and children (2, 3).

Sub-Saharan Africa accounts for 85% of all global maternal deaths, and it has the poorest index of maternal health (3). Furthermore, a considerable proportion of Africa’s maternal deaths occur in Nigeria, which has the fourth highest maternal mortality ratio in the world (3). The problem of maternal mortality in Nigeria is compounded by high infant mortality rate, high fertility rate, and low prevalence contraceptive use (3, 4). In order to improve maternal and child health in Nigeria, the National Population Policy of 1989 was created to foster contraception uptake and reinforce maternal and child health services. The 1989 policy aimed to increase contraceptive uptake by changing attitudes toward contraception and encouraging couples to plan the ideal number of children they would like to have (5). This policy was later revised in 2004 with the additional goals of reducing the occurrence of infant mortality, maternal deaths, and preventing mother-to-child transmission of HIV/AIDS (5). Nonetheless, Nigeria still grapples with high rates of maternal and child mortality. Nigeria’s maternal mortality ratio was 545 deaths per 100,000 live births in 2008 and 576 deaths per 100,000 live births in 2013, while infant mortality rate was 75 per 1,000 live births in 2008 and 69 deaths per 1,000 live births in 2013 (4). Given these somber statistics, it can be inferred that the 1989 and 2004 policies have had limited impact on maternal and child health in Nigeria.

When examining maternal and child health in Nigeria, it is imperative to note religious, educational, and cultural differences between the northern and southern regions that further exacerbate poor health outcomes in the country (6). Nigeria is divided into six geopolitical regions—three in the north and three in the south. The northern region is predominantly Muslim while the south is predominantly Christian. Also, there are variations in literacy levels, age at marriage, and wealth between the regions. Over two-thirds of women and girls in the Northeast and Northwest geopolitical regions are unable to read a complete sentence, and these two geopolitical regions have higher rates of teenage pregnancy and child marriage than the southern geopolitical regions (4, 7). The lower levels of educational attainment, lesser representation in the formal employment sector, residence in remote and hard-to-reach settings, and resulting limited interactions with the health system put many women in northern Nigeria at a disadvantage compared to their counterparts in the south.

Despite these regional differences, Nigerian women and girls in all regions face numerous social and economic barriers that exert negative impact on their health status. For example, statistics pertaining to gender equity in Nigeria are discouraging; Nigeria ranks 125 of 145 countries on the Global Gender Gap, and only 62.8% of Nigerian women and girls (compared 80.2% of boys and men) are literate (4, 8). This glaring disparity between the rights of Nigerian men and women stems from “systemic, pervasive, and deeply entrenched discrimination” against women and girls (6). Hence, Nigeria must seek ways to promote the status of its girls and women, considering the profound consequences of gender empowerment on health.

While there is debate regarding the best indicators for measuring women’s empowerment, it is generally agreed that women’s empowerment can be assessed by their ability to participate in household decision-making that reflects their domestic, economic, and movement autonomies (9–11). Poor empowerment of women has been linked with several indicators of maternal and child health including prenatal care, skilled delivery, postnatal care, and contraceptive use (11–13). Contraceptive use is important in the following areas: preventing fetal, neonatal, and under-5 deaths; reducing maternal mortality; and averting high-risk pregnancy—pregnancy among girls younger than 18 years and women older than 35 years (14, 15). Although it has been noted that women with greater measure of empowerment are more likely to use modern contraception (10, 11), there is limited evidence to support this hypothesis among a population-based sample of Nigerian women (13, 16). Therefore, the purpose of this study was to assess the trend and impact of women’s empowerment on the use of modern contraception among a representative sample of Nigerian women over a decade.

## Materials and Methods

The study used the 2003, 2008, and 2013 Nigeria Demographic and Health Surveys (NDHS). The NDHS is a nationally representative survey that uses a multistage and stratified sampling design to collect information on various areas of population health including maternal health, contraception use, child health, and women’s empowerment. Design and methods of the Demographic and Health Surveys (DHS) are publicly available and have been described (17).

### Measures

The study used engagement in household decision-making to measure women’s empowerment. The DHS questions pertaining to household decision-making are limited to respondents who are married or living with a partner (17). Therefore, single, widowed, separated, and divorced women were excluded from this analysis. Women’s participation in decision-making was assessed by three items, as follows: (1) person who participates in decision pertaining to respondent’s health care; (2) person who decides on large household purchases; and (3) person who decides whether respondent can visit her family or relatives. Women were classified as participating in decision-making if they made the decision alone or jointly with their partner. Otherwise, when the decision was made without the respondent’s input, such women were classified as not being involved in decision-making.

While acknowledging limitations (18), this study adopted the definition of modern contraceptives provided by Hubacher and Trussell (19) “a product or medical procedure that interferes with reproduction from acts of sexual intercourse.” This definition was adopted, given its synergy with NDHS data. Modern contraceptive methods enable couples to have sexual intercourse at any mutually desired time, with diminished risks of pregnancy (18). Forms of modern contraceptive include the following: sterilization, intrauterine devices and systems, subdermal implants, oral contraceptives, condoms, injectables, emergency contraceptive pills, patches, diaphragms and cervical caps, spermicidal agents (gels, foams, creams, suppositories, etc.), vaginal rings, and sponge.

The study was limited to women with the need for contraception, including women who were undecided on the desire for more children, who were unsure of the timing for childbearing, who wanted to limit childbearing, and who wanted children after 2 years following the survey. Otherwise, women who were sterilized, infecund, and wanted children within 2 years of the survey were excluded from this analysis.

The final analytic sample comprised of 3,216 women from the 2003 NDHS, 15,256 women from the 2008 NDHS, and 17,161 women from the 2013 NDHS, making a total of 29,034 respondents across the three surveys.

### Statistical Analyses

The association between women’s empowerment and use of modern contraception was assessed by means of chi-square tests and multivariable logistic regression models controlling for respondent’s religion, age at first marriage or cohabitation, number of children, geopolitical region, wealth index, and highest educational level. Because this is a nationally representative dataset, the analysis was weighted to ensure the generalizability of the data. Data analyses were conducted using the PROC SURVEY command of SAS software, version 9.4 (SAS Institute Inc., Cary, NC, USA). Statistical significance was set at *p* < 0.05.

The NDHS surveys were conducted under the scientific and administrative oversight of National Health Research Ethics Committee of Nigeria and ICF International’s Institutional Review Board. In addition, this secondary data analysis was reviewed and approved as exempt by the Institutional Review Board of the University of Arizona since the DHS contains de-identified data.

## Results

The demographic and decision-making characteristics of the respondents are presented in Table 1. Across the three surveys, there was a higher percentage of women with no education (48.8% in 2003, 45.0% in 2008, and 44.1% in 2013) than women with primary, secondary, or higher educational levels. In addition, at least one in four respondents (34.6% in 2003, 26.7% in 2008, and 25.0% in 2013) reported being younger than 15 years of age at their first cohabitation or marriage. The prevalence of women involved in the decision to visit their relatives or family increased from 42.5% in 2003 to 50.6% in 2013. The proportion of women who participated in decisions concerning large household purchases also increased from 24.3% in 2003 to 41.1% in 2013, while the percentage of women involved in decisions pertaining to their health care increased from 28.4% in 2003 to 41.9% in 2013. The prevalence of modern contraceptive use among the respondents increased marginally from 11.1%% in 2003 to 12.8% in 2013.

**Table 1:** **Demographic and health characteristics of participants**.

	2003 Nigeria Demographic and Health Surveys (NDHS)	2008 NDHS	2013 NDHS

	*n* (%)	*n* (%)	*n* (%)
**Decision to visit family/relatives**			
Self or joint decision	1,441 (42.5)	8,464 (56.3)	8,895 (50.6)
Others	1,775 (57.5)	6,792 (43.7)	8,266 (49.4)
**Healthcare decision**			
Self or joint decision	980 (28.4)	6,750 (45.5)	7,327 (41.9)
Others	2,236 (71.6)	8,506 (54.5)	9,834 (58.1)
**Large household purchase decision**			
Self or joint decision	820 (24.3)	5,967 (39.4)	7,305 (41.1)
Others	2,396 (75.7)	9,289 (60.6)	9,856 (58.9)
**Contraception use**			
Modern	368 (11.1)	1,559 (11.4)	2,196 (12.8)
Traditional/folkloric/no use	2,848 (88.9)	13,697 (88.6)	14,965 (87.2)
**Religion**			
Christian-Catholic	395 (10.6)	1,398 (9.6)	1,454 (8.9)
Islam	1,719 (56.1)	8,488 (54.1)	9,129 (56.9)
Other Christian	1,102 (33.3)	5,370 (36.3)	6,578 (34.2)
**Education**			
No education	1,530 (48.8)	7,456 (45.0)	7,196 (44.1)
Primary	801 (24.8)	3,392 (22.8)	3,769 (20.8)
Secondary	719 (21.9)	3,487 (25.5)	4,857 (27.8)
Higher	166 (4.5)	921 (6.7)	1,339 (7.3)
**Wealth index**			
Poorest	673 (20.3)	3,830 (21.8)	3,341 (20.7)
Poorer	617 (20.0)	3,403 (20.9)	3,468 (20.1)
Middle	635 (19.9)	2,858 (18.2)	3,422 (18.5)
Richer	646 (19.4)	2,705 (18.8)	3,591 (20.0)
Richest	645 (20.4)	2,460 (20.3)	3,339 (20.7)
**Age at first cohabitation or marriage**			
Less than 15 years	1,054 (34.6)	4,221 (26.7)	4,152 (25.0)
15–19 years	1,386 (43.3)	6,851 (43.4)	7,817 (45.5)
20 years and older	776 (22.1)	4,184 (29.9)	5,192 (29.5)
**Geopolitical region**			
North-Central	580 (15.8)	2,551 (12.4)	2,851 (15.4)
Northeast	710 (21.0)	3,200 (14.3)	3,209 (15.8)
Northwest	790 (28.5)	4,081 (29.9)	4,417 (31.2)
Southeast	327 (7.1)	1,218 (9.0)	1,456 (8.3)
South–South	353 (15.4)	1,888 (13.8)	2,347 (11.0)
Southwest	456 (12.2)	2,318 (20.6)	2,881 (18.3)
**Current age**			
15–19 years	239 (8.4)	1,089 (6.7)	1,033 (6.5)
20–29 years	1,210 (38.5)	5,683 (37.0)	6,227 (37.0)
30–39 years	1,003 (30.6)	4,916 (32.6)	5,771 (33.5)
40–49 years	764 (22.5)	3,568 (23.7)	4,130 (23.0)
**Number of children**			
Less than 3	1,330 (41.7)	6,463 (43.1)	7,229 (43.3)
4–6 Children	1,029 (31.2)	5,184 (34.3)	6,148 (35.4)
7 or more	857 (27.1)	3,609 (22.6)	3,784 (21.3)
Total	3,216	15,256	17,161

Table 2 presents results on the association between contraceptive use and women’s characteristics. There was a significant association between all measures of empowerment and use of modern contraceptives. In the 2003 NDHS, there was a significant association between women’s use of contraception and their participation in visiting relatives or family (*p* < 0.05), healthcare decisions (*p* < 0.001), and decision to purchase large household items (*p* < 0.01). In the 2008 and 2013 surveys, there were significant associations between contraceptive use and the following measures of women’s empowerment: visiting families or relatives (*p* < 0.0001), participation in healthcare decision (*p* < 0.0001), and participation in decision-making related to large household purchases (*p* < 0.0001). Across the three surveys, women who participated in decision-making had higher prevalence of contraceptive use compared to women who did not participate. The analysis further showed a significant association between the following demographic characteristics and women’s use of modern contraception: age at first marriage or cohabitation (*p* < 0.0001), education (*p* < 0.0001), parity (*p* < 0.001), and wealth index (*p* < 0.0001). Use of modern contraception was also significantly associated with respondents’ geopolitical region (*p* < 0.0001) and religion (*p* < 0.0001). Women with the following characteristics had a higher prevalence of modern contraceptive use: those residing in southwest and south–south geopolitical regions, aged 20–24 years at their first cohabitation, belonging to other denomination of Christianity than Catholicism, with secondary and higher education, belonging to the richest wealth quintile, and with four to six children.

**Table 2:** **Chi-square test of association between women’s characteristics and contraceptive use**.

	2003 Nigeria Demographic and Health Surveys (NDHS)	2008 NDHS	2013 NDHS

	% of modern contraception	% of modern contraception	% of modern contraception
**Decision to visit family/relatives**			
Self or joint decision	12.9	15.3	19.8
Others	9.7*	6.4***	5.7***
**Healthcare decision**			
Self or joint decision	15.5	16.6	21.0
Others	9.3***	7.1***	6.9***
**Large household purchase decision**			
Self or joint decision	15.0	16.3	22.2
Others	9.8**	8.3***	6.4***
**Geopolitical region**			
North–Central	12.6	12.1	14.1
Northeast	4.0	4.2	3.5
Northwest	4.0	2.8	5.3
South-East	14.9	13.5	13.8
South–South	18.2	17.2	19.1
Southwest	26.3***	24.0***	28.6***
**Age at first cohabitation or marriage**			
Less than 15 years	5.9	5.3	5.1
15–19 years	11.6	9.8	12.0
20–24 years	18.1***	19.3***	20.8***
**Religion**			
Christian-Catholic	16.3	13.7	17.8
Islam	5.6	5.6	6.0
Other Christian	18.7***	19.5***	22.9***
**Education**			
No education	3.1	3.1	2.3
Primary	14.4	13.7	16.9
Secondary	22.0	19.7	22.5
Higher	25.8***	28.5***	28.4***
**Wealth index**			
Poorest	6.0	3.0	1.1
Poorer	3.9	4.3	4.7
Middle	8.8	9.1	11.8
Richer	11.6	15.7	17.5
Richest	24.8***	26.0***	28.9***
**Number of children**			
Less than 3	10.0	10.3	11.1
4–6 Children	14.6	15.3	17.5
7 or more	8.6**	7.8***	8.8***

*p < 0.05; **p < 0.01; ***p < 0.001.

Results of multivariable logistic regression models are presented in Table 3. No significant association was observed between measures of women’s empowerment and use of modern contraception for the 2003 NDHS, but data for 2008 demonstrated significant association between two measures of women’s empowerment and use of modern contraception. Compared to women who were not involved in decision-making, women who participated in the following types of decisions were more likely to use modern contraception: making decisions regarding their own health care (adjusted OR [aOR] = 1.20; 1.04–1.39) and participating in decision to purchase large household items (aOR = 1.15; 1.01–1.31). Similar and stronger associations were observed in the 2013 NDHS, where women who were involved in the decision to visit their family or relatives (aOR = 1.58; 1.36–1.83), pertaining to their health care (aOR = 1.39; 1.22–1.59), and purchasing large household items (aOR = 1.60; 1.40–1.83) had increased odds of using modern contraception than women who were not involved in such decisions.

**Table 3:** **Logistic regression model examining the association between decision-making and contraceptive use (modern vs. traditional or none)**.

	2003	2008	2013

	Adjusted OR (aOR) (95% CI)	aOR (95% CI)	aOR (95% CI)
**Decision to visit family/relatives**			
Self or joint decision vs. others	0.89 (0.63–1.25)	1.17 (1.00–1.36)	**1.58 (1.36**–1.83)***
**Healthcare decision**			
Self or joint decision vs. others	0.99 (0.68–1.43)	**1.20 (1.04**–1.39)*	**1.39 (1.22**–1.59)***
**Large household purchase decision**			
Self or joint decision vs. others	0.91 (0.61–1.36)	**1.15 (1.01**–1.31)*	**1.60 (1.40**–1.83)***

*p < 0.05; ***p < 0.001.

*Model controlled for age at first cohabitation/marriage, number of children, religion, education, wealth status, and geopolitical region*.

*Bold font represents statistically significant association*.

## Discussion

Findings from this study demonstrate a positive association between certain measures of women’s empowerment and modern contraceptive use. Overall, results indicate that measures of Nigerian women’s empowerment improved markedly between 2003 and 2013. This trend is not surprising given recent efforts to improve the status of women and girls in Nigeria, including wider implementation of microfinance programs and implementation of policies that criminalize violence against women (20, 21). Three out of four women who participated in a microfinance program in Zaria, northern Nigeria, reported improved socioeconomic status (20). Also, the percentage of Nigerians with mobile phones has increased tremendously over the past decade; there were 2.38 mobile phones subscriptions per 100 Nigerians in 2003 compared to 82.19 in 2015 (22). Similarly, the prevalence of smartphone and internet use in Nigeria was reported to be 58% in 2015 (23). This is remarkable for a country that had limited reliable telecommunication system two decades ago. It is possible that these rapid increases in access to information technology within the general population may have contributed to the observed improvements in measures of women’s empowerment especially in the realm of mobile health and e-commerce.

Unfortunately, use of modern contraceptives increased only marginally over this 10-year period (11% in 2003 and 13% in 2013). This trend defies the popular model of demographic transition that posits a decrease in fertility rate as a result of modernization (24). Nigeria is the most populous country in sub-Saharan Africa, with an estimated population of 170 million (25). A significant unmet need for contraception is suggested by the country’s 6.0 total fertility rate (TFR), 2.6% annual population growth rate, 0% annual TFR reduction rate between 1970 and 1990, and 0.4% average TFR decrease between 1990 and 2013 (26). Nigeria failed to meet the Millennium Development Goal-5B of universal access to reproductive health services by 2015 (27). Recognition of the unmet need for family planning has prompted the Nigerian government to collaborate with local and international partners in promoting modern contraceptive uptake (13, 28). For instance, with the support of the Reproductive Health Commodity Security Technical Working Group, the federal government of Nigeria developed a strategic plan for 2011–2015 and declared contraceptives free of charge at all public health facilities (29).

In spite of current efforts, many socioeconomic barriers in access to reproductive health services, including use of modern contraceptives, exist for women. Examples of such challenges include geographic barriers and lack of outreach services (30), myths discouraging contraceptive uptake, including the association of contraceptive use with infertility (30) or promiscuity (31), lack of partner support (32), limited knowledge of contraceptives, and missed opportunities for integration of contraceptive use promotion into other existing maternal and child health services (33).

### Strengths and Limitations

The study builds upon previous analyses by using nationally representative surveys as opposed to data collected from few Nigerian cities, thus allowing for nationwide analysis of women’s empowerment and prevalence of use of contraception. Equally, the study used three rounds of Nigerian DHS, which allowed for the examination of progress made over time. However, this study has a few limitations. Since the data used in this study were derived from a cross-sectional survey, it is not possible to show causality between women’s empowerment status and use of modern contraception. Empowerment is also a challenging subject to conceptualize and accurately measure. Although the measures selected for this study have been widely used to characterize women’s empowerment (9–11), it is possible that they do not reflect the true nature of women’s empowerment in the cultural context of Nigeria. In addition, the analysis used only household decision-making indicators as a proxy for women’s empowerment, although this was addressed by adjusting for other possible indicators of women’s empowerment including age at first cohabitation, wealth index, and education. Finally, the analysis did not assess modern contraceptive use by type—i.e., pills, condoms, injectables, or intrauterine device—given the small sample sizes of women who responded to each form of modern contraceptive in the DHS.

## Conclusion

Although measures of Nigerian women’s empowerment increased considerably over a decade, the prevalence of modern contraceptive use remained low and largely unchanged over the 10-year period. Beyond women’s participation in household decision-making, further research is needed to elucidate how measures of women’s empowerment interact with cultural values and health system factors to influence women’s uptake of contraceptives. First, it is important to note that fertility decisions among African unions are influenced by patriarchal values (34). A married woman’s decision to limit or space childbearing might lie with her husband, parents-in-law, or other older relatives, all of whom may not always support the decision. Second, irrespective of a woman’s levels of empowerment, her ability to access and use contraceptives may be influenced by health system barriers. Evidence suggests that such barriers could be significantly reduced through attention to quality issues in contraceptive use promotion (35, 36). With regard to contraceptive use promotion in Nigeria, attention to the following three of Bruce’s six elements of quality (35) may be important:

(1) *choice of methods*: it is important that women are provided with the opportunity to switch methods of contraception as their needs and preferences change over time.
(2) *information, education, and communication*: given the aforementioned misconceptions about contraception (30, 31), service providers have a duty to identify culturally appropriate strategies for communicating factual information about contraception to assist women in making informed decisions to select appropriate methods and to use such methods effectively.
(3) *the technical competence of providers*: program planners can improve technical competence of contraception service providers by conducting needs assessments to deliver culturally competent care that fosters uptake and continued use of contraception by women. Finally, geographic barriers in access to and use of modern contraceptives in Nigeria (4) can be addressed through policies that promote outreach services as well foster over-the-counter sale of contraceptives.

## Author Contributions

IA, CO, and JEE conceptualized the paper. IA, JEE, and CO developed the methodology. IA conducted data analyses and presented the results. IA, CO, JCE, and HD drafted the manuscript. JEE supervised manuscript preparation. JEE reviewed and commented on drafts of the manuscript. All the authors edited drafts and approved the manuscript for submission.

## Conflict of Interest Statement

The authors declare that the research was conducted in the absence of any commercial or financial relationships that could be construed as a potential conflict of interest.
